# Effectiveness of Mental Health First Aid Training in Sweden. A Randomized Controlled Trial with a Six-Month and Two-Year Follow-Up

**DOI:** 10.1371/journal.pone.0100911

**Published:** 2014-06-25

**Authors:** Bengt Svensson, Lars Hansson

**Affiliations:** Department of Health Sciences, Lund University, Lund, Sweden; University of Rochester, United States of America

## Abstract

**Background:**

According to a recent report from the European Brain Council and the European Colleague of Neuropsychopharmacology the one year prevalence of some kind of mental disorder is around 27% among the adult population in Europe. Research has shown a lack of mental health literacy in the population in general and it is thus important to find ways to improve the public's knowledge and skills to provide first hand support to people with mental disorders. Mental Health First Aid (MHFA) is a training program that has shown positive changes in knowledge and helping behavior. This study investigates if MHFA training in a Swedish context provides a sustained improvement in knowledge about mental disorders, a better ability to be helpful in contacts with people who are ill and if it changes attitudes in a positive direction.

**Methods and Findings:**

The introduction of the training program was made in accordance with the constructor's instructions. Participants were mainly public sector employees from a county in the west of Sweden. The study was a randomized controlled trial with an experiment group (n = 199) and a control group (n = 207) placed on a waiting list during a 6-month follow-up. A two-year follow-up was conducted for participants (n = 155) from both the intervention and waiting list group who had completed the training and during the follow-up been in contact with persons with mental disorders. The intervention group improved in knowledge as well as in confidence in providing help for someone in need. The two-year follow-up showed that the improvements were to a great extent maintained.

**Conclusions:**

Mental Health First Aid might raise the level of awareness of mental disorders and have an influence on the number of people who can receive professional treatment for their problems.

## Introduction

Estimations of the burden of mental disorders in Europe indicate that around 27% of the adult population every year suffers from some kind of mental disorder [1], [2]. With a broader inclusion of diagnoses such as childhood and adolescent disorders, mental retardation and dementia the percentages for the population suffering from mental disorders raises to 38.4% [2]. The highest prevalence is reported for anxiety disorders, mood disorders dominated by major depression, somatoform disorders and substance abuse [2]. This leads to a disability burden that counts for between 25 to 30 percent of the total disability adjusted life years lost (DALYs) in Europe [2]. There is also, for a number of different reasons, a high level of unmet health care needs. Studies show that only a minority of all cases receive adequate consultations with professional health care services and that there is a long delay between the recognition of the disorder and the help provided for those who received a consultation [1], [3].

Furthermore, many people are unable to recognize mental illness and have beliefs about causes and treatment that varies greatly from the evidence from mental health research. Stigmatizing attitudes are widespread and hinder recognition and appropriate help-seeking. Although recent studies have shown improvements in mental health literacy among the public there still is a need for more knowledge in the areas of recognition and treatment beliefs for mental disorders [4]. Concerning changes in attitudes there is a trend toward a greater acceptance of professional help seeking but not toward people with mental disorders [5]. In fact, attitudes towards persons with mental disorders have not improved over the last two decades and regarding people with schizophrenia they have deteriorated further [5], [6].

Since the prevalence of mental disorders is high and the possibilities for afflicted persons to get appropriate help in time are limited, the chances of encountering people with mental illness in the community are very likely. It is thus of importance to find ways for improving the general public's knowledge and skills to provide first hand support to people with mental disorders.

Mental Health First Aid training (MHFA) was developed in Australia for improving mental health literacy among the general public and also for giving skills to provide initial help to people in mental health crisis situations and for those with on-going mental health problems. The training includes a 12-hour course where a first aid approach is taught in five steps: 1: Assess risk of suicide and harm, 2. Listen non-judgmentally, 3. Give reassurance and information, 4. Encourage persons to get appropriate professional help, and 5. Encourage self-help strategies. The steps are then applied to depression, anxiety disorders, psychosis and substance use disorder. Furthermore the participants are given specific instructions how to help a suicidal person, a person having a panic attack, a person who has experienced a traumatic event and a psychotic person threatening violence [7].

The effects of MHFA training have been investigated in four randomized controlled trials. The samples investigated include employees in government departments [8], general public in a large rural area in Australia [9], high school teachers, a version adapted for young people [10] and a study comparing the effects of three methods: an internet based course, reading the course book only and a control group [11]. Other research includes one controlled study [12] and eight uncontrolled studies [13]–[20] and these have investigated different populations such as pharmacy students [12], minority groups in Australia [15], [16], football club leaders [17] and officials in rural areas of Australia [18]–[20]. These evaluation studies have shown improvements in mental health knowledge, reduced stigma, increased confidence in providing help and increased provision of help.

The most consistent findings are improvements in knowledge, more confidence in providing help and a reduction of at least some aspects of stigma. There is, however, less support for increased help provided to others [10], [12], [16]–[20]. Three qualitative studies have also been performed, two in Australia and one in the UK [21]–[23], where both instructors and course participants have been investigated. The latter report that they used skills learned and that the content of the course was relevant [21], [22]. All the studies have, with one exception [23], so far been carried out in Australia. It is thus of interest to investigate if the same results can be achieved in another context and to what extent skills are maintained during a two-year follow-up period.

In 2010 the Ministry of Health and Social Affairs in Sweden decided to initiate a major pilot project in order to test and evaluate an implementation of MHFA in Sweden. The present study evaluates the effectiveness of MHFA training for people in public services who have many contacts with the general public. The aims were to investigate if the training program generated a sustained better ability to be helpful in contacts with people who are ill an improvement in knowledge about mental disorders, and if attitudes were changed in a positive direction.

## Methods and Participants

### Trial design

A randomized controlled trial was planned with the control group placed on a waiting list during a 6 month follow-up. The two-year follow up study had a cross-sectional design including participants from the experiment group and the control group who had completed the training and been in contact with a person with a mental disorder during the follow-up period.

### Participants

**Figure 1:**
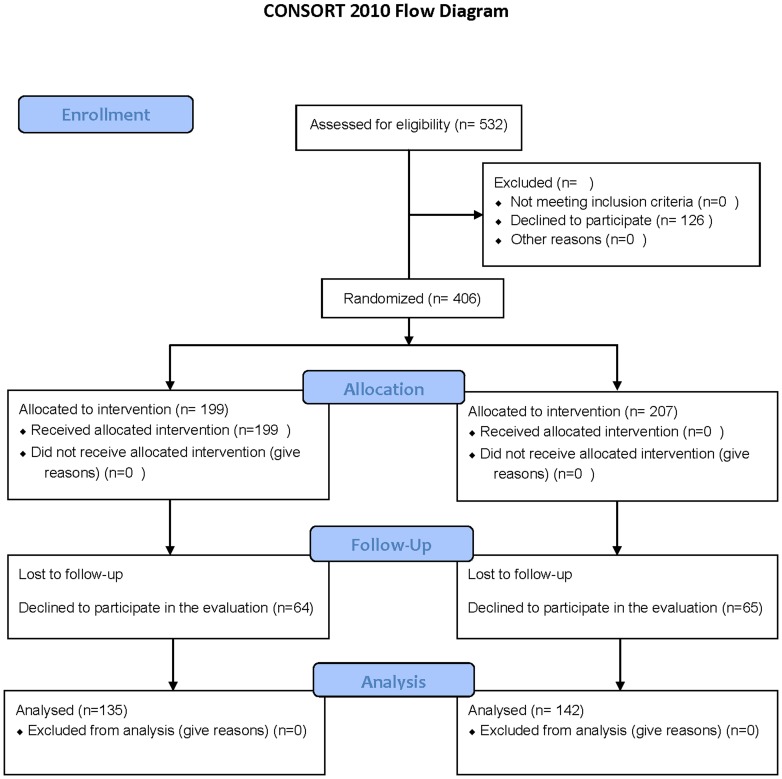
Consort flow diagram.

Staff from the Swedish social insurance agency, employment agencies, social services, schools, police departments, correctional treatment units, rescue services and recreation centers was offered to participate in MHFA training. Everybody who registered for the training was also invited to participate in the evaluation (Figure 1). In the two year follow-up those who completed the training and had given informed consent were invited to participate.

### Setting

The study was conducted in a county in the west of Sweden with about 1.6 million inhabitants and includes the second largest city in the country, Gothenburg, and two medium sized cities, Falköping and Vänersborg. The implementation of the program was coordinated by a local organization in the county called Suicide Prevention In the West (SPIV). Training was given at worksites or in local colleague localities in classes with a maximum of twenty participants.

### Intervention

The introduction of MHFA in Sweden followed the steps described on the Mental Health First Aid Australia's homepage about how to adapt the program for overseas organizations [24]. The host organization for MHFA in Sweden is the National Centre for Suicide Research and Prevention of Mental Ill-Health (NASP) at the Karolinska Institute in Stockholm. An Australian team taught three Swedish main instructors and the complete MHFA program was translated and modified to suit the Swedish context. The main instructors then taught 18 instructors who implemented the training program. All the instructors had experience of mental health work in some form, such as health care staff and volunteers in user organizations. The content of the course has been described here in the introduction and in research literature [7]. All the participants received a MHFA manual in Swedish [25] and attended the twelve hour course, which was equally spread over two days.

### Outcomes

The primary outcome is whether individuals trained in MHFA compared to waiting – list controls improved their readiness to provide actual help to people with mental disorders. Secondary outcomes are if the training lead to more: 1. knowledge about mental disorders and their treatment, 2. knowledge about how to behave and act when with a person with a mental disorder, 3. self-confidence in helping a person with a mental disorder, 4. positive attitudes towards people with mental disorders.

The pre-test assessment was administered by mailed questionnaires with reply envelopes sent to participants in the month prior to the intervention. The follow-up assessment was made in the same way six months after completion of the training. The same self – report questionnaires as in the Australian studies have been used [8], [9]. They cover socio-demographic characteristics of the participant, why he/she were interested in doing the course, self-reported history of mental health problems in participant or family, confidence in providing help, contact with people who have mental health problems in previous 6 months and help offered, recognition of a disorder in vignettes describing a person with depression or schizophrenia, belief about the helpfulness of various interventions for the person described. The beliefs about helpfulness include questions about which professionals might be the right ones to receive help from and what kind of interventions that might help. The maximum score for the depression vignette is six and for the schizophrenia vignette is five. A social distance scale to assess stigmatizing attitudes is also included [26]. The questions in this scale ask how willing respondents would be to (1 =  definitely, 2 =  rather not, 3 =  definitely not) (1) move next door to the person described in the vignette (2) make friends with the person, (3) start work closely with the person, (5) have the person marry into the family. A final question covered if the participant or a family member ever had a problem such as the one described in the vignette. The vignettes describing depression and schizophrenia were given alternately to the participants.

Following accordance with the recommendations from previous evaluators, Anthony Jorm and Betty Kitchener, two scales were added to the present study. Firstly a MHFA knowledge questionnaire that contains two questions from each of the disorder chapters and one question from each of the crisis chapters. It thus includes 16 questions and the alternative responses were: agree, disagree and don't know. The numbers of correct answers are summed, where the alternative “don't know” counts as wrong and the scores can thus vary from 0–16. Secondly, the personal and perceived stigma scale [27], which was originally developed to investigate stigma towards people with depression and comprises 18 items. Nine of the items ask the participant to rate how strongly they personally agree with statements about the person presented in the vignette (depression or schizophrenia) and the other nine questions ask about what they think other people believe about the same issue. Ratings are made on a five-point Likert scale where higher scores indicate less stigmatizing attitudes. The items include whether the described disorder is a real medical illness, to what extent it is under personal control, if the disorder is a sign of weakness, if a person with the disorder is dangerous or/and unpredictable, if to feel shame or/and conceal the illness if stricken by it, avoidance of a person with the disorder and discrimination in terms of “not voting for a politician with the disorder” and “not employing someone with the disorder”. Psychometric testing of the scale has demonstrated acceptable properties concerning internal consistency [27] and test-retest reliability [28]. The internal consistency of the scale for the present sample was 0.76 (Cronbach's alfa).

### Two year follow-up

Outcomes for the two year follow-up were self-reported change in skills in providing support and a self-report covering if the participant had more frequently been providing help to people with mental disorder. A new questionnaire was constructed for this purpose. The initial question asks if the participant has been in contact with a person with a mental disorder since they finished the course. If the answer is “no” they are requested not to answer any further questions and return the questionnaire in the return envelope. If the answer is “yes” they are requested to complete the questionnaire, the items in which were derived from the descriptions in the MHFA manual about how to provide mental health first aid. The questionnaire has two parts where the first part includes nine items covering whether the participant, since completing the MHFA training, had improved their skills in: making contact with a person with mental health problems, taking time and listening non-judgmentally, being aware of how a sad and depressed person communicates, asking if someone has suicidal thoughts, giving information about effective treatment, giving information about how to get right kind of help, recognizing signs of mental disorders, assessing the seriousness when a person is in a crisis and suggesting things that might make someone feel better. The responses are given on a five-point scale ranging from “not at all” to “to a very great extent”. The other part of the questionnaire includes five items and asks the participant whether he/she after the completion of the course has more often: made contact with a person with mental health problems, taken time and listened non-judgmentally, asked someone if he/she has suicidal thoughts, given information about effective help, informed about where adequate help is provided and finally suggested things that might help someone to feel better. For this scale there are four possible responses that range from “not at all” to “on many more occasions”.

All the individuals who had completed the MHFA training and given informed consent for participating in the RCT, both experimental and control group participants, received a questionnaire with attached information about the follow up study by post. Letters to the experiment group were sent during spring 2013 and to the control group, after receiving training 6 months later, during autumn 2013. The questionnaires were coded with the same case number as in the original data set from the RCT. This made it possible to identify participants who had returned the questionnaire and then to investigate differences between those who replied and those who had not. The inclusion criteria for participating in the study were to have completed the MHFA course and to have been in contact with a person with a mental disorder after its completion.

### Randomization and blinding

The course coordinator in the county sent the baseline questionnaires with written information together with a form for giving consent to be randomized to either immediate training or to waiting for about six months. The coordinator created a list of participants who had given informed consent and completed the questionnaires. Each participant was given a specific ID-number on the list, which was then sent to one of the researchers (BS) who performed the randomization procedure by using the Random Integers option at the http://random.org website [29]. The participants where anonymous to the researchers.

### Statistical methods

Intention to treat analyses were performed in the RCT. The multiple imputation module in IBM SPSS was used to impute follow-up data regarding outcomes for participants who completed the baseline assessments but did not participate in the follow-up. In addition to baseline data in the respective outcome variable, gender, age, educational level and belonging to the experimental/control condition was used to predict and impute values for the respective outcome variables at the time of the follow-up.

Time by group differences were analyzed by ANOVA Repeated Measures. Differences in demographic variables were investigated by Chi^2^ - analysis for dichotomous variables and with independent samples T-test for continuous variables. Alpha was set to <0.05. IBM SPSS-statistics 20 was used in all analyses.

Post hoc power analyses were performed using G-power [30]. Power was analyzed using sample size and observed effect sizes as input variables and level of significance was set at p<0.05. Effect sizes were calculated using Cohen's d.

### Ethics statement

The study was approved by the Regional Ethical Review Board at Lund University. Registration no. 2011/4.

## Results

The total number of participants assessed for eligibility was 532. Of these 126 declined to participate. The randomization of the 406 who had given informed consent resulted in an experimental group of 199 participants and a control group with 207 participants. Complete baseline data was collected from both groups. At the six month follow up 64 participants in the experimental group and 65 in the control group had not completed the questionnaires. Demographic data are presented in table 1 (table 1).

**Table 1:** Demographic data for the experimental group (n = 199) and control group (n = 207).

Variables	Experimental group	Control group
Age, mean (sd)	45.6 (10,7)	45.6 (10.3)
	% (n)	% (n)
Women	75.9(151)	78,3(162)
University/college education	74.9(149)	75.4(156)
Born in Sweden	88.4(176)	93.2(193)
Not health care staff	83.3(166)	84.7(175)
Own experience of psychiatric illness	22.7(45)	27.7(57)
Psychiatric illness in family	42.7(85)	44.7(92)
Met someone with mental illness during the last 6 months	85.3(170)	85.9(178)

The majority of the participants were women, middle-aged, with a high level of education and born in Sweden. There were no significant differences between the two groups concerning background characteristics or familiarity with mental health problems.

The primary outcome of the study indicates that MHFA training improved readiness to provide help to people in mental health crisis situations (Table 2). It also improved knowledge about mental disorders and how to act and behave in contact with afflicted persons as well as more confidence in one's own ability to help. Results show, however, a limited effect for change in attitudes. The social distance toward a person with depression and the willingness to become a neighbor with her showed a small but significant positive change. Post hoc power analysis for the significant results showed a power between 0.82 to 0.99.

**Table 2:** Results from analyses.

	Exp. Group (n = 100)		Contr. Group (n = 207)				
Variable	Base line	Follow up	Base line	Follow up	F	*p*	Effect size
	mean(sd)	mean(sd)	mean(sd)	mean(sd)			
**All participants**							
Help offered	2.9(0.9)	3.1(0.9)	2.8(0.9)	2.8(0.9)	5.4	<0.05	0.22
MHFA knowledge	7.2(2.2)	8.7(2.1)	7.3(2.3)	7.3(2.4)	44.9	<0.001	0.63
Confidence in providing help	2.4(0.8)	2.7(0.6)	2.4(0.7)	2.4(0.7)	14.3	<0.01	0.32
**Depression vignette**							
Beliefs about treatment	5.1(1.1)	5.3(1.0)	5.0(1.1)	5.3(1.1)	1.3	n.s*	−0.08
Personal stigma	35.8(5.2)	36.3(4.8)	36.4(4.5)	35.4(5.3)	6.3	<0.05	0.29
Perceived stigma	23.9(7.1)	24.4(6.8)	24.9(6.5)	24.8(6.7)	0.8	n.s*	0.09
Become a neighbour with X	1.4(0.6)	1.2(0.5)	1.2(0.5)	1.3(0.5)	6.5	<0.05	0.34
Become a friend with X	1.5(0.6)	1.4(0.6)	1.3(0.5)	1.4(0.5)	2.7	n.s*	0.19
Become a colleague with X	1.8(0.7)	1.6(0.6)	1.6(0.6)	1.6(0.6)	2.2	n.s*	0.20
X married into family	1.8(0.7)	1.7(0.7)	1.7(0.7)	1.7(0.6)	1.1	n.s*	0.12
**Psychosis vignette**							
Beliefs about treatment	3.7(1.3)	3.8(1.1)	3.7(1.1)	3.8(1.1)	1.2	n.s*	0.04
Personal stigma	33.9(4.9)	33.5(5.2)	33.7(4.5)	33.6(4.7)	1.4	n.s*	0.14
Perceived stigma	22.1(6.1)	22.3(6.1)	21.5(5.9)	22.4(5.8)	2.0	n.s*	−0.17
Become a neighbour with X	1.8(0.6)	1.6(0.5)	1.8(0.6)	1.7(0.6)	0.5	n.s*	0.10
Become a friend with X	1.7(0.7)	1.6(0.6)	1.9(0.7)	1.8(0.6)	0.6	n.s*	0.06
Become a colleague with X	1.9(0.7)	1.7(0.6)	2.0(0.7)	1.9(0.6)	0.5	n.s*	0.08
X married into family	2.2(0.6)	2.0(0.7)	2.3(0.6)	2.2(0.6)	2.7	n.s*	0.22

a Positive effect size indicate positive change in the experimental group, *not significant, The data presented are derived from the intention-to-treat analysis and represent pooled figures from the dataset. Mean values and standard deviations after imputation are presented.

### Two-year follow up

Participants who had given informed consent (n = 406) received questionnaires and reply envelopes. Of these 62 letters were returned to sender, 34 participants reported that they had not been in contact with a person with mental disorder and two reported that they were practicing psychologists and felt it unfair to complete the questionnaires. Thus the final eligible sample consisted of 308 participants and of these 155 completed and returned the questionnaires, giving a response rate of 50%. There were no differences in baseline data between those who responded and those who had not concerning, age, sex, education, self-reported own or family experience of mental disorders, confidence in providing help, actual help offered, belonging to the original experimental or control group or been answering to the vignette for depression or schizophrenia.

Results are presented in Tables 3 and 4. Table 3 shows the percentages of participants who, on a five graded scale, reported the two most positive alternatives of improvements of skills in providing mental health first aid (Table 3.). In Table 4 the percentages of participants who report that they have practiced their skills at “on quite a lot more occasions” or “on many more occasions” are shown (Table 4.).

**Table 3:** Results from the two-year follow- up.

Since I completed MHFA training I have improved my skills in:	%
Making contact with a person with mhp*	47.1
Taking time and listen non-judgmentally	54.9
Knowing what to listen for	48.1
Asking about suicidal thoughts	37.1
Giving information about effective help	46.5
Giving information about where to get effective help	50.3
Recognizing symptoms of mhp*	43.2
Assessing seriousness of mhp*	41.2
Suggesting self-help strategies	39.4

*mental health problems.

Percentages of participants who report that their skills in providing mental health first aid have improved “to a great extent” or “to a very great extent”. (n = 155).

**Table 4:** Results from the two-year follow-up.

Since I completed the MHFA training I have more often:	%
Made contact with a person with mhp*	36.3
Stayed and listened non-judgmentally to a person with mhp*	51.0
Asked about suicidal thoughts	32.9
Given information about effective help	49.0
Given information about where to get effective help	45.8
Suggested self-help strategies	48.4

Percentages of participants who report that they have practiced their skills “on quite a lot more occasions” or “on many more occasions”. (n = 155).

*mental health problems.

The results show that the Mental Health First Aid training after two years still have a notable impact on the awareness of mental health and its treatment. It also leads to behavior change in terms of a readiness to engage more in contacts with people with mental health problems.

The most difficult part of the mental health first aid intervention appears to be to ask questions about suicidal thoughts and the easiest is to listen non-judgmentally. The proportion of participants, who report that they have not improved skills in asking about suicidal ideation, is 17.4% and for those who had not practiced this skill is 26.5%. However, the study provides no information about the extent to which participants actually met people where the question would be relevant.

## Discussion

The aims of the study were to investigate the effectiveness of MHFA-training and the sustainability of gained knowledge and skills. This is to our knowledge the first randomized controlled trial performed outside Australia and the first with a follow-up period longer than six months. It can also be seen as a test of the proposed methodology for implementing the MHFA-training by following the steps for adapting the program to overseas organizations [24].

The results support the findings from previous RCTs of MHFA [8]–[11]. The intervention group improved more in knowledge about what to do and how to behave in contacts with persons in different kinds of mental health crisis, as well as in confidence in providing help and actually giving help to someone in need. However, changes in attitudes were infrequent and mainly concerned depression. Beliefs about treatment did not improve significantly, which has been reported in earlier studies [8]–[11]. The latter could be explained by a ceiling effect because the participants in both the intervention and control group had already good knowledge about treatment alternatives at base-line.

The two-year follow up indicated that the improvements gained during the course were maintained for a longer period of time. This is supported by an earlier qualitative study performed 19–21 months post-training where information was collected by gathering stories about providing mental health first aid [21]. The researchers reported that a majority of the participants had been in situations concerning mental health and that the MHFA-course had enabled them to intervene with a more positive outcome than would have otherwise been possible. In the present study a majority also reported that they had encountered people with mental disorders. Among those who were invited to complete the present two-year follow up study only 12 percent of the participants reported that they not had been in contact with someone with mental health problems. This confirms the findings of a high prevalence of mental illness in the population [2] and underlines the need for improved mental health literacy among the public [31]. According to the self-reported improvements in skills in providing important aspects of mental health first aid, 40–55% of the participants perceived that they achieved this “to a great extent” or “to a very great extent”. This includes issues of great importance such as giving information about effective treatment and where this is available. One of the main targets for the mental health first aid is to stimulate people to seek professional help and the presentation of such information might provide motivation and facilitate doing so. A major question about the MHFA-training is whether it not only changes knowledge but also helping behavior. This study gives some support for such skills being used. Almost 50 percent report that they had provided both information about effective help and where to get it “on quite a lot more occasions” or “on many more occasions”. This does not, however, necessarily entail that more people with mental illness seek professional help. This question is yet to be answered.

There are limitations in the study. Being as the implementation of MHFA was directed to public authorities with responsibility for functions in the society concerning support and services the sample is not representative of the general public. The majorities of the participants had a high level of education and were women and generalizations must be made with caution. On the other hand, most of them had a basic education where mental health issues are included in the curriculum (e.g. social workers, human resource managers, employment officers). It is also quite common with in-house training in a number of fields for staff in these authorities. A great majority also reported that they had met someone with mental illness during the last six months, one fifth had own experience of mental disorder and 40% had experience from their families. From the participant's educational background and personal experiences it could be hypothesized that the MHFA-training could not bring anything new to this group and that the results should thus be negative. Since this not was the case it could be said that the training program withstood a difficult test.

The attrition rate between base-line and six months follow-up was rather high. Whether the causes for this are negative experiences of the MHFA course or something else is difficult to ascertain. All the participants had attended the whole course and if they were dissatisfied or felt it was of no use they could have just left. It is most likely that the attrition rate is due to the everyday puzzle for full time working women with families. Completing questionnaires from a distant university may probably have low priority for this group. In order to control for missing data an intention-to-treat approach was used. Since participants in the intervention group actually received the intervention, techniques such as last observation carried forward was not considered appropriate. It has also been shown that multiple imputations are more adequate and perform better than traditional methods when dealing with missing data and was therefore chosen [32].

In the two-year follow-up it was possible to get information from 155 of the 406 participants. Even if there were no differences between responders and non-responders concerning background characteristics there may be a selection bias in the sample, however, it is not possible to know in which direction the bias can be.
